# Feasibility of intratracheal tracheostomy sealing: anatomical adaptation and biocompatibility of a second-generation prototype in cadaveric and porcine models

**DOI:** 10.1038/s41598-025-21640-z

**Published:** 2025-10-28

**Authors:** Rasmus Ellerup Kraghede, Louise Winding Nielsen, Karen Juelsgaard Christiansen, Stig Dyrskog, Alexander Emil Kaspersen, Esben Beier Gynning, Jens Toft Væsel, Michael Pedersen, J. Michael Hasenkam

**Affiliations:** 1 Department of Intensive Care, Aarhus University Hospital, Aarhus, Denmark; 2 Department of Cardiothoracic and Vascular Surgery, Aarhus University Hospital, Aarhus, Denmark; 3 Department of Clinical Medicine, Faculty of Health, Aarhus University, Palle Juul-Jensens Boulevard 69 - F303, 8200 Aarhus, Denmark; 4 Comparative Medicine Lab, Aarhus University Hospital, Aarhus, Denmark

**Keywords:** Respiratory insufficiency, Tracheostomy, Decannulation failure, Cough efficacy, Mechanical ventilators, Pulmonary rehabilitation, Respiratory signs and symptoms, Implants, Biomedical materials, Respiratory distress syndrome

## Abstract

After decannulation, air leak through the tracheostoma can impair cough and voice. We evaluated a second-generation temporary intratracheal sealing disc that seals the stoma from within. Two preclinical sub-studies were performed: Sub-study 1 cadaveric deployment to assess anatomical fit, seal integrity, and removability; and Sub-study 2, a 7-day porcine implantation with anatomical compatibility and blinded histology. In cadavers, the sealing disc maintained airway patency and sealed the stoma, withstanding peak intratracheal pressures up to 180 cmH_2_O; removal through a near-healed opening required approximately 5 N traction. In the porcine model, no airway obstruction or clinical distress was observed, and histology (Phase Two) showed no excess inflammation or foreign body reaction versus sham. The sealing disc provided intraluminal sealing of the tracheostomy with feasible insertion and removal characteristics and a favorable short-term tissue response in preclinical models. These findings support clinical feasibility testing of this approach to maintain an intact airway dynamic during early post-decannulation healing.

**Supplementary Information:**

The online version contains supplementary material available at 10.1038/s41598-025-21640-z.

## Introduction

Tracheostomy has proven beneficial in patients requiring prolonged mechanical ventilation and has been widely established as a common practice in advanced critical care^1–3^. Once weaning from mechanical ventilation is successful, the tracheostomy tube is removed, and spontaneous healing of the tracheostomy is expected to occur within 7–14 days^4,5^. During this period, a bandage is commonly applied over the tracheostomy to reduce air leakage and limit the passage of secretion through the stoma^5^.

### Problem

Despite its widespread use, the conventional bandage is associated with significant air leakage and reduced cough efficacy, which may impede effective clearance of airway secretions, thus compromising pulmonary function^6,7^. Even a single cough can loosen or displace the occlusive dressing, creating an air leak that diverts flow through the tracheostomy and reduces subglottic pressure. Although routine dressings suffice for most patients, a vulnerable subgroup – those unable to reliably manually occlude the stoma due to neuromuscular or cognitive limitations, and who have low cough peak flow and high secretion burden – are disproportionately affected^8^. In these patients, the resulting ineffective cough promotes secretion retention and impaired O_2_ and CO_2_ exchange. Such conditions may precipitate atelectasis and pneumonia, potentially leading to decannulation failure, necessitating reintubation^9^. Decannulation failure is a frequent occurrence, with an incidence ranging between 4 and 41%^8,10,11^. Yet, research efforts to mitigate this issue remain sparse^12,13^. Efficient sealing of the tracheostomy immediately after decannulation will restore physiological airway anatomy and respiratory mechanics, instantly^7^. This may contribute to preserving effective cough strength, minimizing the incidence of atelectasis, and maintaining optimal oxygenation across the alveolar membrane^9^. Furthermore, good cough strength improves successful weaning from ventilator treatment, minimizes decannulation failure, and may shorten in-hospital stays^14^.

### Intratracheal tracheostomy sealing

A concept aimed at addressing these clinical challenges has already been subjected to preliminary studies^15–17^. An intensive care nurse (Karen Juelsgaard Christiansen) has invented an intraluminar sealing disc, which can be inserted through the open stoma, providing immediate and effective closure of the tracheostomy wound, and can be removed in toto after healing of the tracheostoma has been accomplished. The tracheostomy sealing disc has been improved in a second-generation prototype after the initial studies.

### Hypothesis and aim

We hypothesized that sufficient closure of the tracheostoma using a second-generation tracheostomy sealing disc is feasible without causing airway obstruction or eliciting a foreign body reaction. This study aimed to assess the anatomical adaptation and biocompatibility of a second-generation tracheostomy sealing disc by investigating optimized sealing disc positioning, removal dynamics, adjacent tissue composition, and foreign body reaction.

## Material and methods

The feasibility of an earlier version of the tracheostomy sealing disc has previously been evaluated in a preclinical setting and tested in a clinical crossover study^15,17^. A prototype of a second-generation tracheostomy sealing disc was produced by Technolution A/S, Hørsholm, Denmark, and used in the study. To evaluate the sealing disc’s performance, the present study was structured into two sub-studies. Sub-study 1 assessed anatomical adaptation, including deployment, sealing effectiveness, and removal in a human cadaver model. Sub-study 2 evaluated biocompatibility in an animal model, focusing on local tissue response and foreign body reaction towards the sealing disc material.

### The tracheostomy sealing disc

**Fig. 1:**
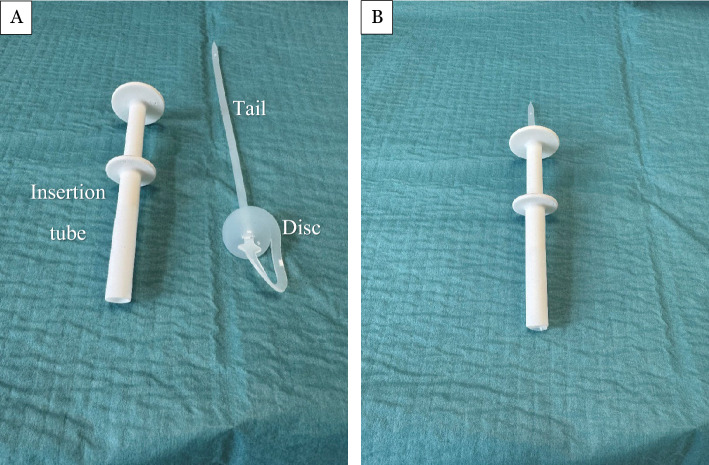
Second-generation sealing disc (medical-grade silicone; USP Class VI). (**a**) The sealing disc consists of a disc placed intraluminally in the trachea, sealing a tracheostomy; the tail exits the stoma for fixation and permits removal by unwinding into a single silicone strand. The insertion tube consists of a hollow tube and a piston (See Methods for insertion/removal details.). (**b**) The sealing disc is placed in the insertion tube, ready for insertion. Pushing the piston into the insertion tube will deploy the sealing disc.

**Fig. 2:**
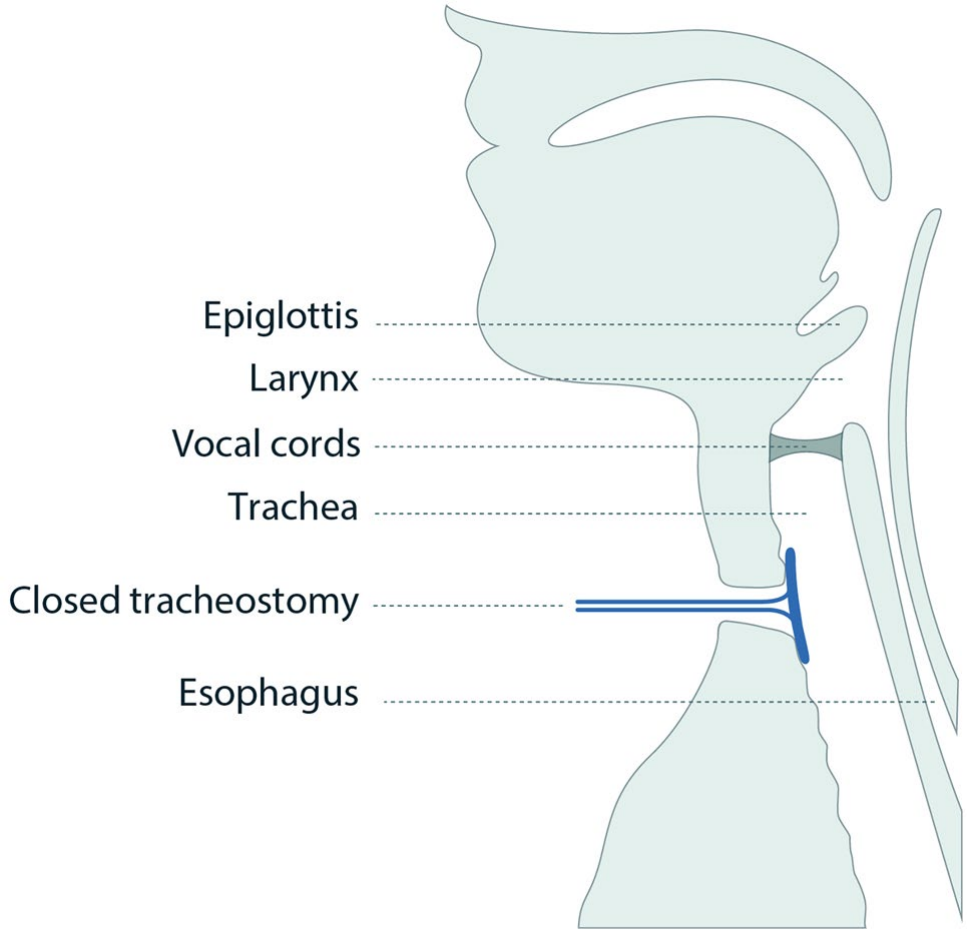
Schematic representation of the intended intratracheal placement of the sealing disc in a clinical setting. *Reproduced from* *Kraghede* et al., *Biomedicines (2024)*, https://doi.org/10.3390/biomedicines12040852*, under the terms of the Creative Commons Attribution (CC BY) license.*

The second-generation sealing disc, as seen in Fig. 1, consisted of a circular disc with a slender tail, cut from a solid block of medical-grade solid silicone rubber, 50 durometer 71-MED (Industry Specifications: USP Class 6, CFR 177.2600, ASTM D2000). To facilitate insertion, a dedicated insertion tube was developed. The sealing disc was folded and loaded into the tube; upon insertion, the piston advanced the sealing disc into the tracheal lumen, after which the insertion tube was withdrawn, leaving the sealing disc in its correct position. A slight pull on the “tail” of the sealing disc ensured that the sealing disc aligned with the tracheal lumen lining. Once deployed, the sealing disc resided within the tracheal lumen, sealing the tracheostomy from the inside. Figure 2 shows a schematic representation of the intended intratracheal placement of the sealing disc in a modelled clinical setting. The sealing disc featured predetermined weak points, enabling it to unwind into a single silicone strand when traction was applied to the tail. Following wound healing around the tail, the sealing disc could be removed in one piece through the residual opening by pulling the tail.

### Sub-study 1: anatomical adaptation in human cadavers

Sub-study 1 was performed at the Department of Biomedicine, Aarhus University, Aarhus, Denmark. No ethical approval was required, since the cadavers were to be used for research. The study was acknowledged by Aarhus University, Aarhus, Denmark, as being in accordance with the rules of research on deceased donors. Two newly deceased formalin-submerged human donors with no visible abnormalities or damage in the anatomy of the neck and upper airways were selected for evaluation of the anatomical adaptation of the tracheostomy sealing disc.

#### Deployment of the sealing disc

**Fig. 3:**
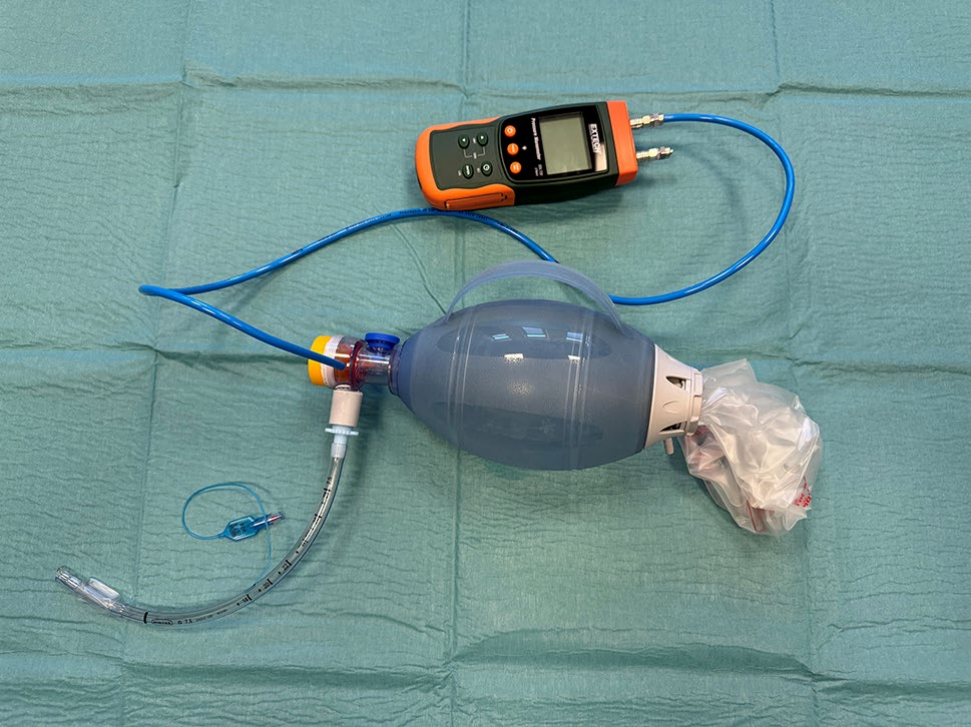
Manometric setup for cadaver testing (Sub-study 1[*n* = 2] cadavers): cuffed endotracheal tube connected to a self-inflating bag with an inline manometer; exhalation valve occluded with a yellow cap, high-pressure air release occluded with a blue cap, to create a closed system. Pressures recorded during bag inflations to assess seal integrity.

The first human cadaver was intubated with a 7.0 mm endotracheal tube (Smiths Medical ASD, Inc., Minneapolis, Minnesota, USA), pressuring the cuff maximally. An 8 mm biopsy punch (KAI Medical, Solingen, Germany) was used to create a circular hole to provide a close representation of a fibrous tracheostomy channel. The compressed tracheostomy sealing disc was inserted through the tracheostomy using the application tube, and re-expansion on the intraluminal side of the trachea was visually verified by endoscopic visualization using a bronchoscope (aScope™ 4 Broncho Regular 5.0/2.2, Ambu A/S, Ballerup, Denmark) through an endotracheal tube. The airways were then pressurised via the endotracheal tube using a self-inflating resuscitator (Oval Silicone Resuscitator, Ambu A/S, Ballerup, Denmark) that had been modified to allow airway pressurization whilst blocking exhalation. The resuscitator was equipped with a pressure gauge (Pressure Manometer SDL720, Extech Instruments, Nashua, New Hampshire, USA) to monitor air pressure loss through the sealed tracheostomy. This setup can be seen in Fig. 3. The resuscitator was fully manually compressed three times, and the pressure was measured continuously for 60 s for qualitative assessment of any air leakage through the tracheostomy. Pressure was plotted as a function of time using SAS Enterprise Guide software, version 7.1 (SAS Institute, Cary, NC, USA).

#### Removal of the sealing disc

In the second cadaver, a test of the sealing disc’s extractability was performed through a simulated healed tracheostomy wound created using a percutaneous dilational tracheostomy kit (Smiths Medical ASD, Inc., Minneapolis, Minnesota, USA), dilating to 3–5 mm with a 14 Fr dilator. Similar to retrograde intubation^18^, the sealing disc was inserted in a retrograde fashion through the endotracheal tube by advancing a guidewire through the tracheostomy from the intratracheal side and then inserting the sealing disc using a suture. The sealing disc was removed by manually pulling the tail of the sealing disc through the simulated healed tracheostoma and the force required was recorded with a newton meter (RS Pro 111–3690, Allied Electronics, 7151 Jack Newell Blvd. S. Fort Worth, Texas, TX 76,118 USA) connected to the tail of the sealing disc.

#### Endpoints and outcome measures

The human cadaver model served primarily as a qualitative assessment of the sealing disc’s mechanical performance and its compatibility with human anatomy. We evaluated the sealing efficacy of the sealing disc by measuring the air pressure (cmH_2_O) in the trachea while the sealing disc sealed the simulated tracheostomy. Further, we measured the force (Newton) required to unwind and remove the sealing disc through the tracheostoma. Lastly, the overall compatibility, ease of placement, and removal of the sealing disc were qualitatively evaluated by the research team.

### Sub-study 2: biocompatibility in a porcine model

Sub-study 2 was performed at the animal experimental laboratory of the Department of Clinical Medicine, Aarhus University, Aarhus, Denmark. The study was approved by the Ministry of Food, Agriculture and Fisheries of Denmark (permission ID: 2021–15-0201–00,998) and was conducted in accordance with Danish and European regulations for animal experiments as well as the ARRIVE guidelines^19–21^. Twenty female 60-kg Yorkshire-Landrace pigs were included in the study. Sub-study 2 consisted of two phases. Phase One was conducted to refine our surgical approach, optimize deployment and extraction of the sealing disc, and improve airway dynamic testing and tissue harvesting techniques. Due to the animals’ behavior and environmental conditions, infections frequently developed around the sealing disc when it was used to seal a tracheostomy. As a result, it was not possible to distinguish between tissue responses caused by the sealing disc itself and those resulting from infection. This demonstrated the need for two phases of the animal study.

**Fig. 4:**
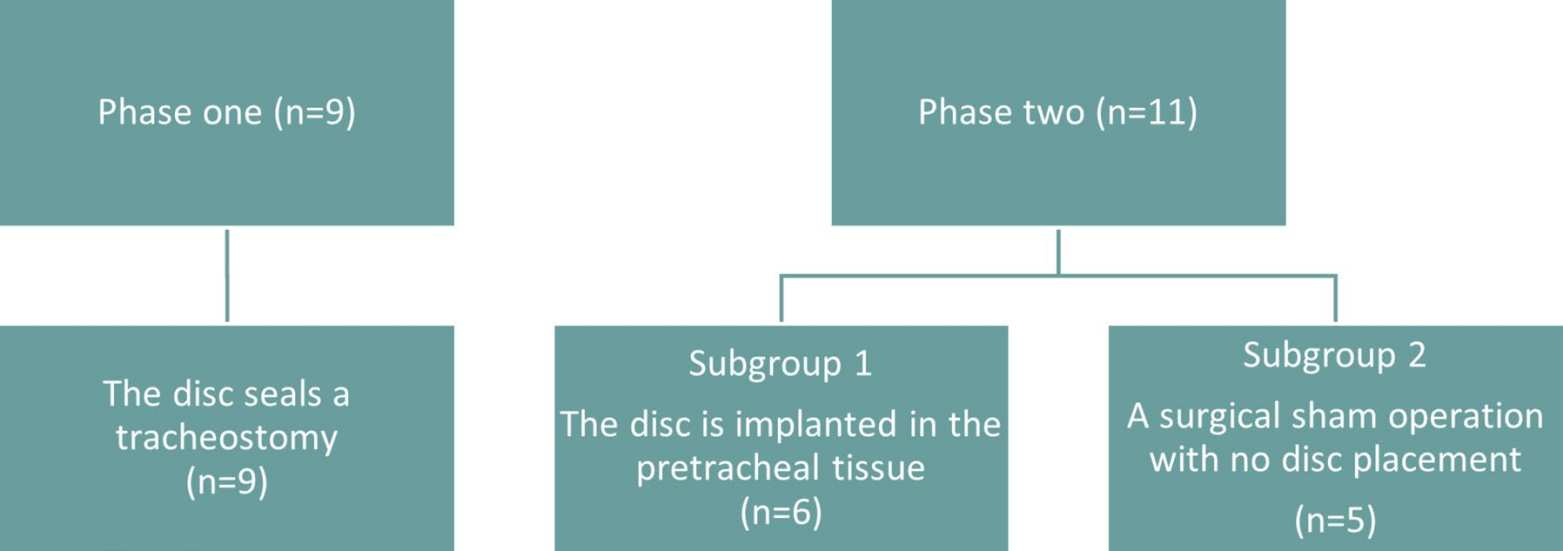
Sub-study 2 (porcine) flowchart: allocation to Phase One and Phase Two, device vs sham; 7-day follow-up; endpoints (airway safety, device position, blinded histology). Group sizes: Phase One: [*n* = 9]. Phase Two: sealing disc [*n* = 6], sham [*n* = 5].

Phase One was a prospective cohort study in which the pigs were monitored for any discomfort while the sealing disc was in place. It furthermore allowed for the evaluation of possible degradation of the sealing disc. Phase Two was a single-blinded, controlled study evaluating tissue response when having the sealing disc implanted. The evaluators assessing tissue response were blinded to group allocation. Figure 4 illustrates the distribution of animals across Sub-study 2 and the focus of each phase.

#### Sample size rationale

Phase Two of Sub-study 2 was conceived as a feasibility, concordance-based comparison of blinded qualitative histology between Subgroup 1 (sealing disc implanted) and Subgroup 2 (sham). In a previous study of a similar design, the histological analysis showed no foreign body reaction or necrosis across multiple high-power fields^15^. Moreover, the sealing disc is manufactured from medical-grade silicone meeting USP Class VI biocompatibility criteria and is widely used in implantable devices^22,23^; together with the temporary implantation in this study, only minimal tissue reaction is anticipated. Accordingly, the categorical nature of the endpoint, standardized sampling, multiple fields evaluated per animal, and the expectation of minimal reaction warrant a modest cohort (5–6 animals per group) as sufficient to support histologic assessment and still adhere to the 3Rs (use of the minimum number of animals consistent with the scientific objective)^21^.

#### Surgical procedure

All pigs underwent general anaesthesia using propofol (5 mg/kg/h) and fentanyl (12.5 μg/kg/h) and were mechanically ventilated. Handling, medication, transportation, and housing of the pigs at our research facilities have previously been described in detail^24,25^. To expose the trachea, a 5–6 cm sagittal incision was made in the midline over the trachea. Pre-tracheal tissue was divided, and the trachea was exposed.

**Fig. 5:**
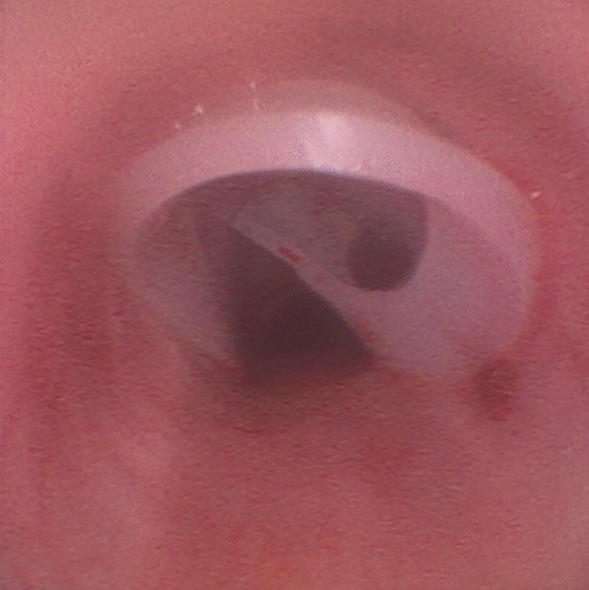
Bronchoscopic view (Sub-study 2, Phase One [*n* = 9]): Sealing disc intraluminally sealing a tracheostomy.

In Phase One, the 8 mm biopsy punch was once again used to form a circular tracheostomy of a fixed size at the 3rd and 4th tracheal rings. The sealing disc was placed through the tracheostomy, sealing the tracheostomy. Placement was confirmed with the bronchoscope via the endotracheal tube as seen in Fig. 5. The tail of the sealing disc was tunnelled and fixated to the subdermal side of the skin with suture (Monosyn 3–0, Ethicon). This adapted fixation avoided penetration of the tail through the skin, which would be an open access for infection, not expected in a human clinical setting. The wound was closed in two layers, with a running suture for the subcutis (Vicryl 3–0, Ethicon) and an intracutaneous suture for the skin (Monosyn 3–0, Ethicon). In Phase Two, subgroup 1, the sealing disc was placed between the trachea and the pre-tracheal tissue, with the trachea remaining intact. The tail of the sealing disc was fixated to the surrounding tissue, and the wound was closed in two layers. Subgroup 2 in Phase Two of the study was a sham control, meaning that no sealing disc was placed in the pigs. The surgical procedure was a sham procedure identical to the procedure for subgroup 1, but with no sealing disc placement. In Phase Two, the trachea remained intact without a tracheostomy in both subgroups. Following surgery, the animals were monitored by facility caretakers twice daily and engaged in cognitive and physical training. Any signs of suffering or distress observed in the animals were reported by the caretakers to the research team, who took action if necessary.

#### Sample collection

All pigs were euthanized after 7 days of observation using 100 mg/kg pentobarbital. Tracheal and adjacent tissue containing the surgical area and all tissue in contact with the tracheostomy sealing disc were excised en bloc from all animals immediately following euthanasia. Tissue specimens were promptly fixated in 10% formalin, paraffin-embedded, sliced into 5 µm-thick cross-sections, and stained with haematoxylin and eosin. Histological slides were scanned using the NanoZoomer S360 (Hamamatsu Photonics Deutschland GmbH, Herrsching am Ammersee, Germany) and evaluated in NDP.view2 (version 2.9.29; Hamamatsu Photonics K. K., Hamamatsu City, Shizuoka Pref., Japan).

#### Endpoints and outcome measures

Phase One: Primary endpoint was airway safety, defined as the absence of stridor or other signs of airway obstruction. Secondary endpoints included animal welfare (participating in daily exercises, eating/drinking behavior, overall well-being), sealing disc performance at follow-up – correct intratracheal position and maintenance of the sealing disc’s structure – and feasibility of removal through the healed tracheostomy without adjunct instruments.

Phase Two: The primary endpoint was histological tissue response to the implanted sealing disc compared with sham surgery. Histology was assessed by a blinded senior pathologist using predefined qualitative descriptors (present/absent; none/mild/moderate/marked) for acute inflammatory infiltrates, necrosis, and foreign body reaction with excessive granulation response. Data were summarized descriptively due to the feasibility design and small cohorts.

## Results

### Sub-study 1: anatomical adaptation in human cadavers

**Fig. 6:**
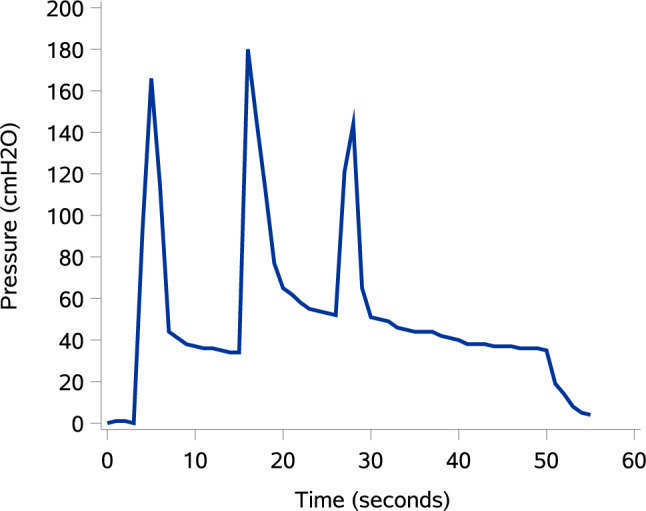
Intratracheal pressure over time during cadaver stress-testing (Sub-study 1, [*n* = 2] cadavers): three manual bag inflations with peak pressures 140–180 cmH_2_O and subsequent stable plateaus (30–65 cmH_2_O).

Bronchoscopy revealed that the sealing disc fitted nicely along the tracheal wall and sealed the tracheostomy. The lumen of the trachea was unobstructed, allowing for free air passage. Figure 6 shows the pressure in the trachea as a function of time. Sealing the tracheostomy wound with the sealing disc allowed for air pressure peaks between 140 and 180 cm H_2_O in the trachea. Following each of the three times of full inflation of the ventilation bag, the airway pressure remained stable at 30–45, 50–65, and 35–50 cmH_2_O, respectively. No air leak was observed through the tracheostomy.

Furthermore, in preliminary force testing on “healed” tracheostomies, the sealing disc withstood a threshold of 4 Newton of pulling force before deformation and removal of the sealing disc began. The peak force was 5 Newton while removing it from the tracheostomy wound.

### Sub-study 2: biocompatibility in a porcine model– phase one

Among the nine pigs included in Phase One, none demonstrated stridor or any other signs of airway obstruction during the seven-day follow-up period. All study animals engaged actively in daily exercise, displayed normal eating and drinking behaviours, and exhibited signs of overall well-being. Six of the nine pigs had a correctly positioned sealing disc that effectively sealed the tracheostoma at follow-up. An example of this is shown in Supplementary Fig. S1. The sealing disc maintained its structure and was unaffected seven days after deployment. The sealing disc was removed through the healed tracheostomy without difficulty or complications. Supplementary Video S1 shows an example of the removal process, which required approximately 11 s and did not necessitate additional instruments. Within the remaining three pigs, the sealing disc had started the removal process during follow-up due to a combination of tight sutures holding the tail of the sealing disc and the animal’s movements. Macroscopic and microscopic signs of infection were present at the time of sealing extraction in eight of the nine pigs, though no signs of distress were identified in these animals.

### Sub-study 2: biocompatibility in a porcine model– phase two

**Fig. 7:**
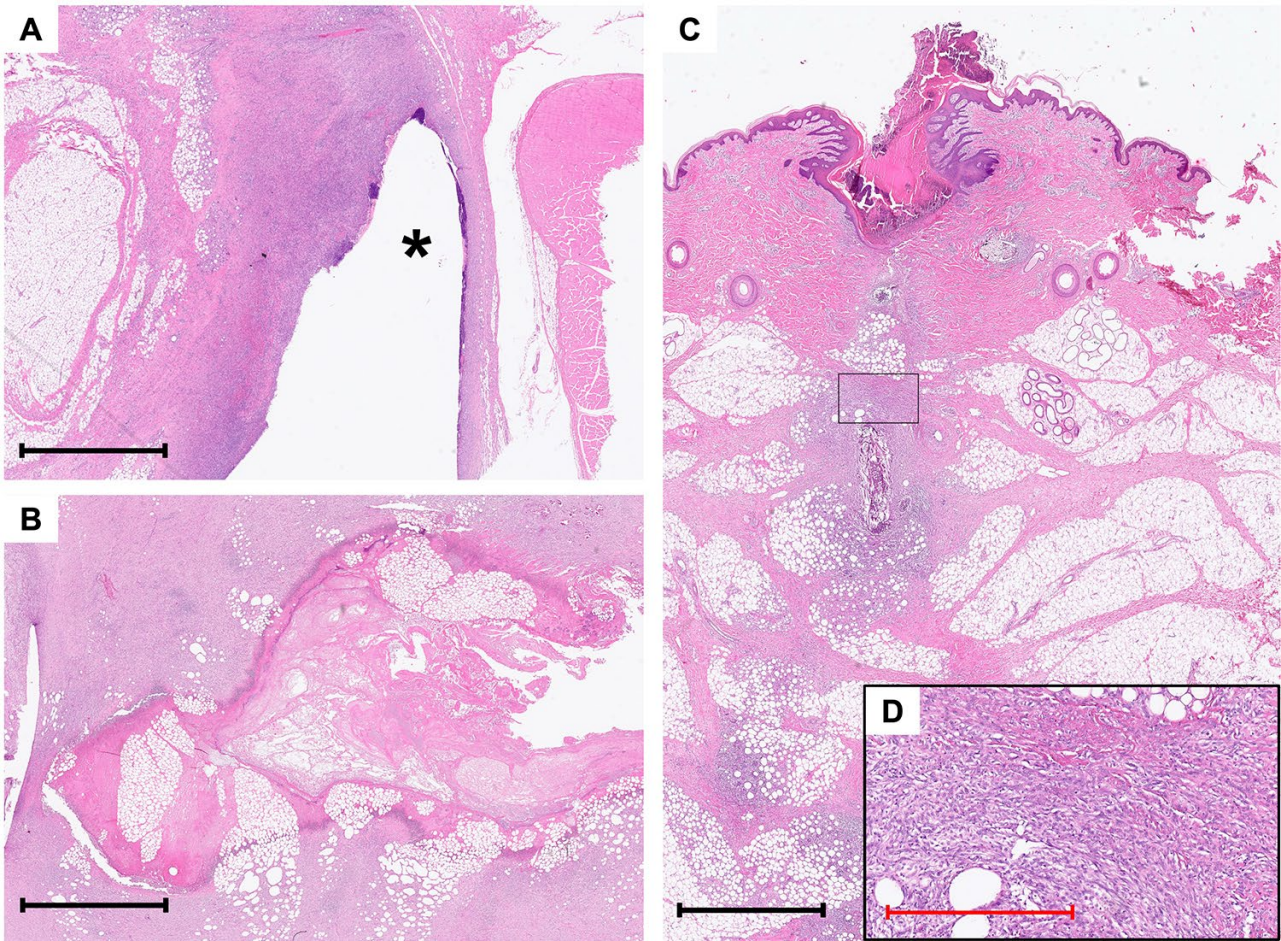
Hematoxylin and eosin-stained sections of the surrounding tissue to the trachea from Sub-study 2, Phase Two (sealing disc [*n* = 6], sham [*n* = 5]). (**a**) Subcutaneous tissue adjacent to a lumen created by the sealing disc in (asterisk) in a pig from Phase Two, subgroup 1 (sealing disc [*n* = 6]). (**b**) Tissue necrosis from a macroscopically infected pig of Phase One. (**c**) Overview of the histological impact of surgical trauma from a sham control pig (Phase Two, sham [*n* = 5]), displaying similar microscopic signs of acute inflammation and development of granulation tissue. (**d**) Higher magnification of the same specimen, showing similar features as in image (a). *Black ruler: 2 mm; red ruler: 500 μm.*

The qualitative histological assessment in Phase Two revealed evidence of healing with similar presence of acute inflammation, angiogenesis, and granulation tissue formation in all animals, regardless of whether the sealing disc had been implanted, as seen in Fig. 7. Signs of chronic inflammation were observed sporadically, with lymphocytes, macrophages, and foreign body giant cells present but without an apparent spatial relation to the location of the sealing disc. No chronic fibrosis had formed. No microscopic tissue necrosis was observed in the animals and in none of the histological slides did we identify clear cellular signs of excessive foreign body reaction.

## Discussion

In this study, we successfully produced a sealing disc with the ability to seal off a tracheostomy wound without compromising free air passage in the airways. In Sub-study 1, the sealing disc was able to withstand extreme physiological pressures momentarily, equal to a cough^26,27^. The sealing disc was able to maintain continuous supraphysiological airway pressure for 20 s, with the pressure dropping less than 1 cmH_2_O per second (from 51 to 35 cmH_2_O) and with no sign of air leakage through the tracheostomy channel. Given that physiological airway pressures are substantially lower, this level of sealing performance should be considered sufficient for clinical use^28^. Furthermore, the sealing disc was easily removed through the simulation of an almost healed tracheostomy with forces harmless to human anatomy^29,30^, yet with a threshold that possibly could prevent accidental removal to a large extent. In Sub-study 2, the sealing disc maintained its form after seven days of in vivo testing. In addition, the sealing disc did not cause any histologically verified foreign body reaction in the tissue around the implanted sealing disc. The inflammatory response in the sham operation animals was overall the same as that where the sealing disc was implanted pre-tracheally. The three cases of unintended removal in Phase One were most likely caused by the animal’s daily playful interaction with the caretakers and a higher level of physical activity compared to what a patient in a hospital bed would display after prolonged ventilator treatment. Nevertheless, it seems advisable to have a flexible spring-like material to compensate for the tension of the tail of the sealing disc for future development.

Previous studies have performed animal experiments with a preliminary sealing disc prototype^15^, as well as a clinical acute study with a handheld prototype of a sealing disc^17^, and concluded that this method works in an acute setting. With this study, we have shown that the method of sealing the tracheostomy is feasible and safe in an in vivo porcine model for a seven-day period. In concert with our previous studies, the present study’s evaluation of anatomical compatibility and tissue response provides a sufficient basis for a clinical feasibility study to evaluate the safety of deployment and removal of the sealing disc, as well as the seal’s impact on airway dynamics and stoma closure.

### Limitations

In this study, we used cadavers that had been submerged in a formalin solution for approximately 48 h. This fixes the anatomical structures, and they become rigid, leaving us with a representation of human anatomy despite a stiffer tissue representation. The downside of this model is that the lungs are filled with liquid, so the high air pressures we produced in Sub-study 1 can only be maintained shortly before the air distributes to other liquid-filled lung sections of the lungs or causes pneumothorax. For this reason, we did not extend the leakage tests over longer periods with physiological air pressure, since progressive air redistribution in the liquid-filled lungs could be a confounder for interpretable results.

Force testing of sealing disc removal was limited to two samples and should therefore be interpreted with caution. Nevertheless, these results indicate that removal does not require excessive force, which is more important than the exact numerical values.

Infection presented a challenge during Phase One of the porcine model. It is presumed that pathogens responsible for the infection migrated from the airways into the peritracheal tissue along the sealing disc. This observation suggests that a fresh surgical intervention involving a foreign body in a non-sterile environment increases the risk of infection.

An additional limitation of this study is that airflow and stoma closure were not investigated. These functional outcomes are more meaningful to perform in a human clinical setting. Performing such studies in a preclinical setting would have very limited translational value. Systematic evaluation in future clinical studies is beyond the scope of this feasibility work.

This preclinical feasibility study – intended as a precursor to forthcoming clinical feasibility studies – used the smallest possible cohorts without a formal power calculation. Results are presented descriptively; therefore, precision and generalizability are limited, and findings should be interpreted as exploratory.

### Perspective

While most patients achieve uncomplicated closure with external dressings, a subgroup experiences delayed airtight closure and difficulty manually occluding the stoma to speak or cough, leading to secretion retention and reduced voice quality. The interval between decannulation and airtight stoma closure remains understudied. Limited reports suggest clinical challenges during this period, especially in the subgroup described above^4,6^. Temporary intraluminal sealing is intended for this subgroup to restore intact airway dynamics during the healing phase. From our earlier human clinical acute study^17^, we have already demonstrated clearly improved airflow and vocalization in patients with a sealed tracheostomy. We hypothesize that sealing may improve airway dynamics, airway sanitation, and blood oxygenation in the lungs compared with conventional spontaneous healing of the tracheostomy. Temporary sealing is also envisioned to accelerate stoma closure by reducing mucus flow across the tract while it heals. After sealing disc removal, the smaller residual stoma is intended to be covered with an occlusive dressing and is expected to pose fewer air-leak and secretion-related issues than the immediate post-decannulation opening. We further hypothesize that sealing the tracheostomy wound could enable more effective pulmonary physiotherapy with continuous positive airway pressure and positive expiratory pressure therapy immediately after decannulation^31,32^. This could have a positive impact on airway secretion, airway sanitation, and cough strength. Ultimately, sealing the tracheostomy could lead to expedited patient recovery, reduced decannulation failure rate, and reduced in-hospital stay^14,33^, which could have a substantial impact on health care costs for tracheostomized patients worldwide. These outcomes will be assessed prospectively.

## Conclusion

The second-generation sealing disc was successfully inserted and sufficiently closed the tracheostomy wound. There were no signs of airway obstruction, and the sealing disc was easily removed after seven days of sealing the tracheostomy. The materials used caused no excessive macroscopic or histologic foreign body reaction. We propose that this sealing disc could be further developed into a device for use in clinical practice, specifically in the intensive care unit, to support patient recovery following tracheostomy. Further research, including clinical testing in patients, is required before broad-scale use of our technology can be introduced.

## Supplementary Information

Supplementary Information 1.

Supplementary Information 2.

Supplementary Video 1.

## Data Availability

The datasets generated and analysed during the current study are available from the corresponding author on reasonable request.
