# Supplementary figures and images for: Feasibility of intratracheal tracheostomy sealing: anatomical adaptation and biocompatibility of a second-generation prototype in cadaveric and porcine models
Source: Sci Rep. 2025 Oct 28;15:37685. doi: 10.1038/s41598-025-21640-z (PMC12568967; doi:10.1038/s41598-025-21640-z)

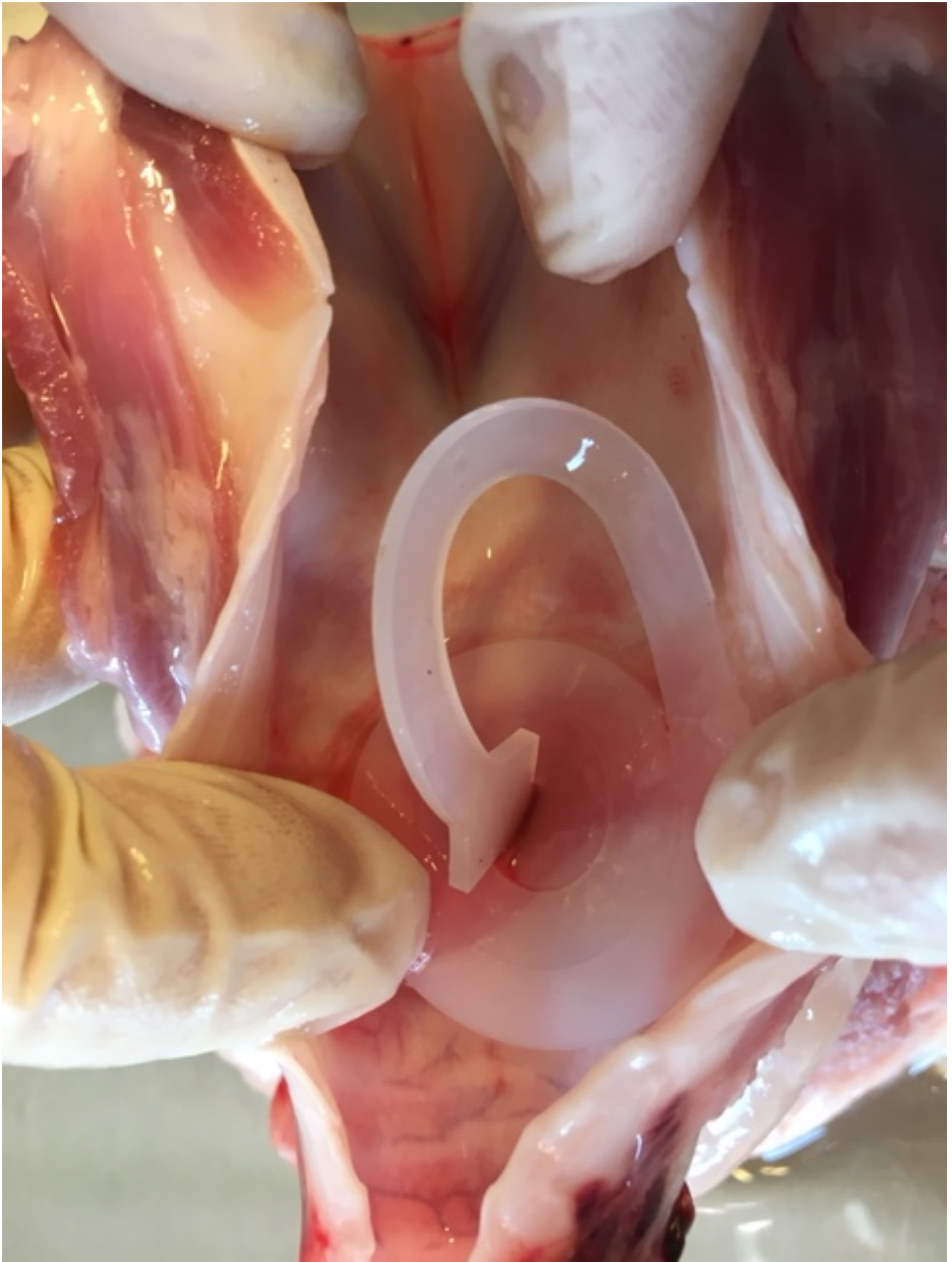

Supplement: Supplementary file 1 — Supplementary Information 1. [file 41598_2025_21640_MOESM1_ESM.pdf]
