# Supplementary material for: Feasibility of intratracheal tracheostomy sealing: anatomical adaptation and biocompatibility of a second-generation prototype in cadaveric and porcine models
Source: Sci Rep. 2025 Oct 28;15:37685. doi: 10.1038/s41598-025-21640-z (PMC12568967; doi:10.1038/s41598-025-21640-z)
Supplement: Supplementary file 2 — Supplementary Information 2. [file 41598_2025_21640_MOESM2_ESM.docx]

# **SUPPLEMENTARY FILE LEGENDS**

**Supplementary Figure S1.** A porcine trachea cut open at the posterior wall, revealing the sealing disc in place against the anterior tracheal wall. Picture taken on the seventh postoperative day

**Supplementary Video S1.** The video shows a porcine trachea cut open at the posterior wall and the sealing disc being removed through a tracheostomy. The disc has sealed the tracheostomy for 7 days.
